# Cardiovascular responses to leg muscle loading during head-down tilt at rest and after dynamic exercises

**DOI:** 10.1038/s41598-019-39360-6

**Published:** 2019-02-26

**Authors:** Cristiano Alessandro, Amirehsan Sarabadani Tafreshi, Robert Riener

**Affiliations:** 1 Northwestern University, Feinberg School of Medicine, Department of Physiology, Chicago, USA; 2 ETH Zurich, Sensory-Motor Systems Lab, Institute of Robotics and Intelligent Systems, Department of Health Sciences and Technology, Zurich, Switzerland

## Abstract

The physiological processes underlying hemodynamic homeostasis can be modulated by muscle activity and gravitational loading. The effects of leg muscle activity on cardiovascular regulation have been observed during orthostatic stress. Here, we evaluated such effects during head-down tilt (HDT). In this posture, the gravitational gradient along the body is different than in upright position, leading to increased central blood volume and reduced venous pooling. We compared the cardiovascular signals obtained with and without leg muscle loading during HDT in healthy human subjects, both at rest and during recovery from leg-press exercises using a robotic device. Further, we compared such cardiovascular responses to those obtained during upright position. Loading leg muscles during HDT at rest led to significantly higher values of arterial blood pressure than without muscle loading, and restored systolic values to those observed during upright posture. Maintaining muscle loading post-exercise altered the short-term cardiovascular responses, but not the values of the signals five minutes after the exercise. These results suggest that leg muscle activity modulates cardiovascular regulation during HDT. This modulation should therefore be considered when interpreting cardiovascular responses to conditions that affect both gravity loading and muscle activity, for example bed rest or microgravity.

## Introduction

**Figure 1:**
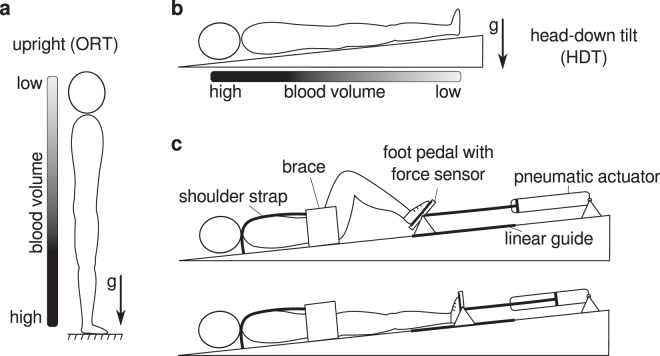
Upright and head-down tilt postures, and schematic representation of the robotic leg-press device. During upright posture (**a**) blood tends to shift towards the lower part of the body under the effect of gravity. This displacement of blood is regulated by physiological mechanisms that accomplish hemodynamic homeostasis, which are modulated by the activity of leg muscles engaged in maintaining the upright position. During head-down tilt (**b**) gravity leads to a displacement of blood towards the upper part of the body, and since there is no weight bearing, leg muscles are inactive. The robotic device MARCOS (**c**) allowed us to apply leg muscle loading and perform leg-press exercises during HDT. The device provides simulated ground reaction force by means of pneumatic actuators that oppose leg extension movements, and maintains the desired force using force feedback loops that receive force measurements from the foot pedals. Additional details on this device are described in Sec. Methods.

The autonomic nervous system can accomplish hemodynamic homeostasis under a variety of conditions, including during physical exercise and gravitational stress. During orthostatic challenges, for example, sympathetic vasoconstrictor activity maintains blood pressure, avoiding postural hypotension and syncope^1,2^. While such activity is primarily regulated by baroreflexes, previous research demonstrated that the contraction of leg muscles modulates cardiovascular regulation^3–7^. Similarly, previous research showed that leg muscle activity influences the physiological processes involved in recovery from physical training during orthostasis^8,9^. Whether these findings hold true also in the absence of orthostatic stress is still unclear. A posture like head-down tilt (HDT, i.e. subjects lie supine on a head-down tilted platform), for example, increases central blood volume (CBV), stimulates baroreceptors and facilitates venous return (Fig. 1a,b). These factors modulate the mechanisms of hemodynamic homeostasis^10–13^, and so may also modify the role of leg muscle activity on cardiovascular function.

Head-down tilt is extensively used in the context of space physiology to simulate the effects of microgravity on the cardiovascular system^14–17^. Experiments have shown that short-term exposure to HDT reduces heart-rate, mean arterial pressure, and leg blood volume, and increases cardiac output^15,18–20^. In the long term, HDT induces a decrease of plasma volume, reduces baroreflex sensitivity, and may ultimately cause orthostatic intolerance^16^. Compared to upright posture, however, HDT (and in general any supine position) not only removes orthostatic stress, but also unloads leg muscles typically used in maintaining the upright position, e.g. hip, knee and ankle extensors (Fig. 1a,b). The observed cardiovascular responses may therefore be due to a combination of these two factors. To better interpret these cardiovascular responses, it is therefore important to determine the potential role of leg muscle activity on cardiovascular function. Establishing this role may also contribute to a better understanding of the cardiovascular deconditioning caused by microgravity^21–26^, and of the physiological processes involved in recovery from physical exercises during space missions^27,28^.

In this study, we evaluated the effects of leg muscle loading during HDT. To do so, we developed a head-down tilted platform equipped with a robotic leg-press device^29^ (Fig. 1c). Such a device provides a simulated ground reaction force that opposes leg extension. Hence, in order to counteract the resistance of the device, the user should mainly recruit muscles such as gluteus maximus, the quadriceps, the hamstrings, and gastrocnemius^30^. Thus, this system allowed us for the first time to load, during HDT, muscles that are typically used to maintain the upright position against gravity (often referred to as “antigravity muscles”). We collected systolic, diastolic, mean blood pressure, pulse blood pressure and heart rate from healthy volunteers lying on the tilted platform, at rest as well as immediately after they performed bouts of leg-press exercises. We performed these recordings both with muscle loading (HDT-ML), when subjects maintained their legs extended against the resistance of the device, and without muscle loading (HDT-noML), when subjects maintained their legs extended against no external resistance (i.e. the device did not apply any force). Additionally, we recorded the cardiovascular signals during orthostatic stress (i.e. upright posture at rest), allowing us to compare these signals to the cardiovascular responses observed during HDT.

Counteracting the resistance of the leg-press device during HDT modified mainly the cardiovascular signals at rest, and only minimally affected their short-term dynamics after exercises. The blood pressure values obtained with muscle loading at rest were significantly higher than those obtained without muscle loading, and were close to those observed during orthostatic stress. This observation suggests that the cardiovascular responses to HDT are partially due to a reduction of leg muscle activity compared to upright posture, and not only to the different gravitational gradient. These results are consistent with the hypothesis that leg muscle activity modulates cardiovascular functions in the absence of orthostatic stress.

## Results

### Head-down tilt without leg muscle loading

**Figure 2:**
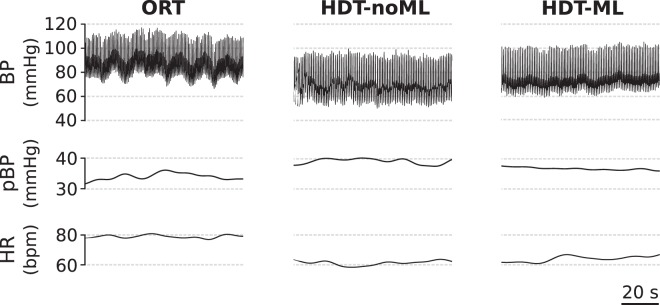
Cardiovascular signals at rest for a representative subject. During head-down tilt without leg muscle loading (HDT-noML), the overall BP trace (i.e. systolic and diastolic) was lower than during both orthostatic stress (ORT) and head-down tilt with muscle loading (HDT-ML). In the HDT posture, pulse BP was slightly higher and heart rate was lower than during ORT, and they were minimally affected by leg muscle loading.

We first evaluated the cardiovascular responses to head-down tilt without leg muscle loading (HDT-noML) at rest (i.e. subjects lay on the tilted platform without performing any movement and maintained leg extension against no external resistance). Figure 2 illustrates an example of the cardiovascular signals for a representative subject. HDT-noML caused a reduction of the entire blood pressure (BP) trace (i.e. lower values of both systolic and diastolic BP), a minimal increase of pulse BP (pBP), and a very evident decrease of heart rate (HR).

**Figure 3:**
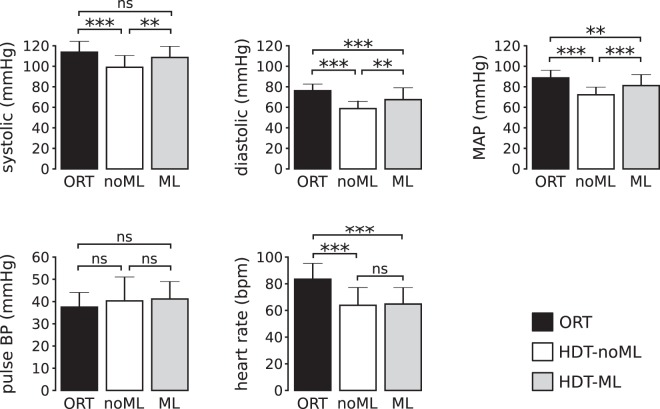
Cardiovascular signals at rest for all subjects. During head-down tilt without muscle loading (HDT-noML), systolic BP, diastolic BP, mean arterial pressure and heart-rate were lower than during orthostatic stress (ORT). During head-down tilt with muscle loading (HDT-ML), systolic BP, diastolic BP and mean arterial pressure were higher than during HDT-noML, while pulse BP and heart rate were not different from HDT-noML. Note that systolic BP during HDT-ML was not different from ORT. Data are presented as mean ± s.d. across subjects. N = 16, 17, 16 subjects contributed to the bars ORT, HDT-noML, and HDT-ML respectively for all signals (see Sec. Methods). ***p < 0.001, **p < 0.01, *p < 0.05.

These results were consistent across subjects, as shown in Fig. 3. During HDT-noML, systolic (sBP), diastolic (dBP) and mean blood pressure (MAP) were significantly lower than during orthostatic stress (ORT; p_*sBP*_ < 0.001; p_*dBP*_ < 0.001; p_*MAP*_ < 0.001); pulse BP was slightly higher but not significantly different (p = 0.321); and HR was significantly lower (p < 0.001).

### Leg muscle loading during HDT at rest

Compared to orthostatic stress (ORT), HDT without muscle loading (HDT-noML) both altered the gravitational gradient along the body and removed weight bearing, hence unloading leg muscles. To determine the relative contributions of these two changes on the observed physiological measures, we asked subjects to maintain leg extension against the resistance of a robotic leg-press device (see Sec. Methods) during HDT, hence loading leg muscles (condition HDT-ML). We then compared the obtained cardiovascular responses to those observed during ORT and HDT-noML. Such analyses were instrumental to: (1) evaluate the influence of leg muscle activity on cardiovascular functions during HDT, and (2) assess the extent to which the results obtained during HDT-noML (section above) were driven by the reduction of leg muscle activity compared to ORT.

Opposing the resistance of the leg-press device increased arterial blood pressure during HDT at rest, and restored similar values to those observed during orthostasis, as illustrated in Figs 2 and 3. The HDT-ML condition induced significantly higher values of systolic BP (p = 0.002), diastolic BP (p = 0.002) and mean arterial pressure (p < 0.001) compared with those elicited by HDT-noML. In contrast, it resulted in non-significantly different values of pulse PB (p = 1) and heart rate (p = 0.92) compared to HDT-noML. Such higher values of systolic BP were not significantly different from ORT (p = 0.132), unlike what was observed without muscle loading. On the other hand, the HDT-ML values of diastolic BP, MAP and heart rate were significantly lower (p_*dBP*_ < 0.001; p_*MAP*_ = 0.003; p_*HR*_ < 0.001), and pulse BP was not significantly different from ORT (p = 0.175), consistent with what was observed during HDT-noML. These results suggest that leg muscle activity modulates the mechanisms of blood pressure regulation during HDT at rest, and contributed to the BP differences between ORT and HDT-noML described above.

### Muscle loading during HDT after leg-press exercise

Leg muscle activity may also affect the cardiovascular processes involved in recovery from dynamic exercises during HDT. We investigated this issue by comparing the cardiovascular signals obtained after performing dynamic leg-press exercises in two scenarios: (1) when subjects maintained leg extension against the resistance of the leg-press machine before and after the exercise bouts (muscle loading condition, HDT-ML), and (2) when the device did not produce any force and therefore subjects did not need to overcome any external resistance (no muscle loading, HDT-noML).

**Figure 4:**
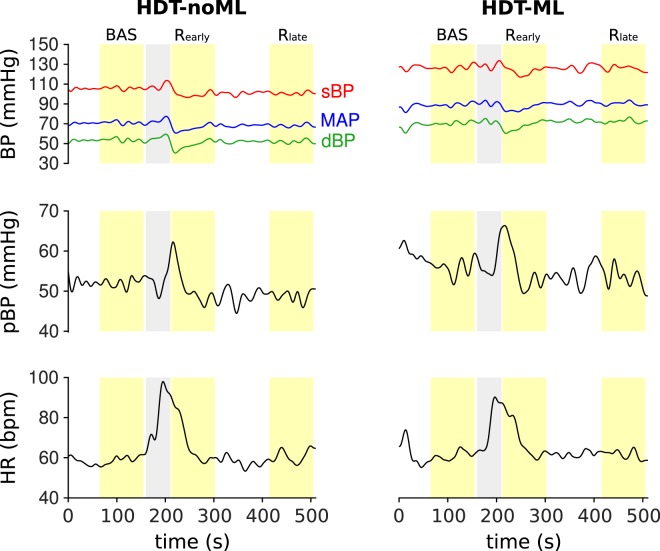
Cardiovascular signals before, during and after dynamic leg-press exercises for one subject. The trends of the signals obtained with leg muscle loading (HDT-ML) are qualitatively similar to those obtained without muscle loading (HDT-noML). All the signals exhibit very clear transient dynamics shortly after the exercise bout, and then they stabilize at later time points. Gray band: preparation to exercise (i.e. the subject waits with their legs flexed) and exercise bout. Yellow bands: baseline (BAS), early recovery (R_*early*_, i.e. first 90 seconds after the end of the exercise bout), late recovery (R_*late*_, i.e. 3.5 to 5 minutes after the end of the exercise bout). During baseline and recovery the subject keeps their legs extended. See Sec. Methods for details on the protocol.

Figure 4 illustrates an example of the the cardiovascular signals obtained without and with muscle loading for one subject. During the first 90 s of recovery (early recovery, R*early*), the signals exhibit clear transient dynamics, which disappear within 200 s when cardiovascular processes have stabilized. During early recovery, diastolic BP and MAP feature well evident undershoots with respect to baseline (i.e. value of the signals before the exercise bout, BAS), and then they stabilize at values slightly lower than baseline for this subject. Systolic BP follows a similar trend, but with a less clear minimum peak. Pulse BP keeps increasing at the beginning of the recovery phase, and then it returns to values similar to baseline. Finally, heart rate slowly decreases towards baseline levels starting from the high values reached during exercise. These trends were qualitatively similar across loading conditions.

In order to quantify the transient dynamics observed during early recovery, we defined the following features:

- Minimum values of sBP, dBP and MAP with respect to baseline (ΔsBP_*min*_, ΔdBP_*min*_, ΔMAP_*min*_), to quantify the initial undershoots of these signals;
- Maximum value of pBP with respect to baseline (ΔpBP_*max*_), to quantify its overshoot;
- Maximum value of HR with respect to baseline (ΔHR_*max*_), to quantify the maximum value of this signal at the beginning of the recovery phase.

**Figure 5:**
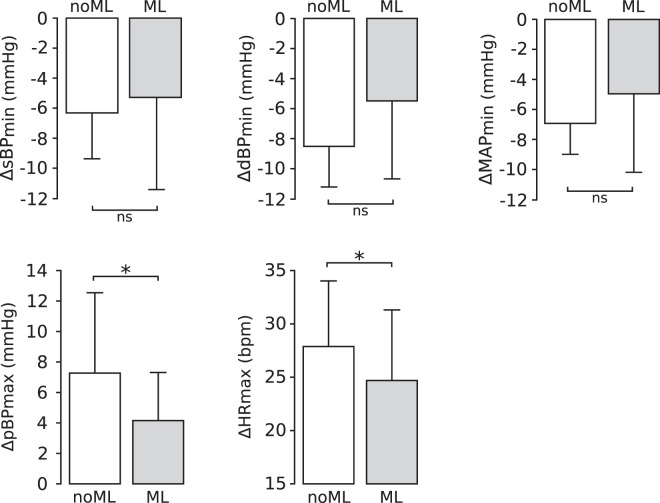
Cardiovascular responses during early recovery with and without leg muscle loading for all subjects. Muscle loading resulted in significantly lower pulse BP peaks and heart rate maxima, and did not affect systolic, diastolic and mean arterial pressure. Data are presented as mean ± s.d. across subjects. N = 17 and 16 subjects contributed to the bars HDT-noML and HDT-ML respectively (for all signals). *p < 0.05.

The comparison between muscle loading conditions in terms of these features is presented in Fig. 5. The undershoots of sBP and MAP obtained with muscle loading were not significantly different from those observed without muscle loading (ΔsBP_*min*_: p = 0.429; ΔMAP_*min*_: p = 0.153). On the other hand, muscle loading resulted in significantly lower values of ΔHR_*max*_ than those obtained without muscle loading (p = 0.024). Finally, results were at the limit of statistical significance for the features ΔdBP_*min*_ (p = 0.050) and ΔpBP_*max*_ (p = 0.047). These results suggest that leg muscle activity minimally affects the transient dynamics of the cardiovascular signals in recovery from leg-press exercises during HDT.

**Figure 6:**
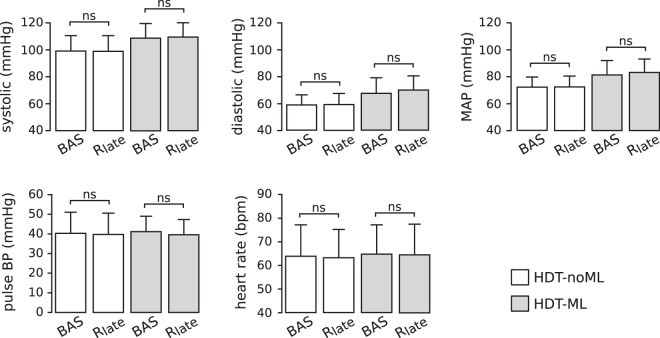
Cardiovascular signals before leg-press exercises and during late recovery with and without leg muscle loading for all subjects. At the end of the recovery phase (R_*late*_), the mean values of all signals returned to values that were similar to those observed before the exercise bouts (baseline, BAS), both with and without leg muscle loading (HDT-ML and HDT-noML respectively). Data are presented as mean ± s.d. across subjects. N = 17, 17, 16 and 16 subjects contributed to the bars BAS (HDT-noML), R_*late*_ (HDT-noML), BAS (HDT-ML), R_*late*_ (HDT-ML) respectively.

The values of the cardiovascular signals observed during baseline and late recovery are illustrated in Fig. 6. As already discussed above, the values of systolic BP, diastolic BP and MAP obtained with leg muscle loading were higher than those obtained without muscle loading. However, there were no significant differences between the values observed during late recovery and those observed at baseline for any signal, both with muscle loading (p = 1 for all signals) and without muscle loading (p = 1 for all signals). This result is further confirmed by the non-significant interaction term (p_*sBP*_ = 0.799; p_*dBP*_ = 0.57; p_*MAP*_ = 0.623; p_*pBP*_ = 0.75; p_*HR*_ = 0.88) between the muscle loading condition (HDT-ML or HDT-noML) and the phase of the experimental session (baseline or R_*late*_) in our statistical model (see Sec. Methods). In other words, independently of muscle loading condition, within 5 minutes after the exercise bouts, all the signals returned to values similar to those observed during baseline.

## Discussion

We investigated the influence of leg muscles loading on cardiovascular regulation in the absence of orthostatic stress both at rest and after dynamic leg-press exercises. When subjects had to counteract the force exerted by the leg-press device during HDT at rest, their BP level increased, re-establishing orthostatic values of systolic BP. This condition of muscle loading affected also the transient dynamics of the signals shortly after exercise, with a small reduction of pulse BP and HR peaks relative to baseline, but not the values reached 5 minutes after the end of the exercise bouts. Taken together, these results suggest that leg muscles activity contributes to cardiovascular regulation in the absence of orthostatic stress.

The main limitation of this study is that we did not measure muscle activity. Although we were unable to determine the specific muscles that were active while maintaining leg extension against the resistance of the device, such a condition should mainly involve gluteus maximus, the quadriceps, the hamstrings, and gastrocnemius. Previous studies from our lab confirm that MARCOS engages these muscle groups^30^. More importantly, the activation of these muscles when subjects had to oppose the resistance of the device (HDT-ML) was most probably higher than when subjects did not have to oppose any resistance (HDT-noML). We are therefore confident that we can interpret the different cardiovascular responses to the HDT-noML and the HDT-ML conditions as deriving from these different levels of muscle activation.

Changing posture from upright to HDT (without muscle loading, HDT-noML) caused a reduction of HR, dBP, sBP and MAP. These responses are consistent with previous observations^15,18,20^, and result from the displacement of fluids from the lower to the upper part of the body. Such a redistribution of body fluids statically stimulates the carotid baroreceptors and increases central blood volume (CBV), thus evoking baroreflex activity that leads to peripheral vasodilation and a reduction of HR. These factors contribute to a sustained decrease of dBP, sBP and MAP at the heart level^18,31,32^. The physiological mechanisms underlying these responses are still under investigation. However, it has been suggested that increasing CBV may alter the characteristics of the baroreflexes, leading to lower MAP and HR compared to upright posture^33–35^.

Two types of neural mechanisms might underlie the influence of muscle activity on cardiovascular regulation: central commands^36,37^, hypothetically originating in motor cortex, and the exercise pressor reflex^38,39^, directly evoked by muscle contraction via activation of mechanoreceptors and metaboreceptors. Both of these mechanisms produce a temporary alteration of the carotid baroreflexes^40^, causing the simultaneous increase of BP and of sympathetic discharge and HR. Accordingly, leg muscle loading during HDT at rest resulted in significantly higher values of sBP, dBP and MAP compared to HDT without muscle loading at rest. However there was no change in HR, which may result from a preferential involvement of the exercise pressor reflex^41^ and from a reduced sensitivity of the carotid-cardiac baroreflex due to the elevated CBV^12,33,34^. The minimal alteration of pulse BP and HR after muscle loading is consistent with the expected responses to static exercises, which evoke small changes in cardiac output and a large increase in MAP.

Performing physical exercises elicited well defined after-effects on the cardiovascular signals. The physiological processes that occur in such a recovery period are distinct from those that characterize the resting and the exercising states, and are currently an active topic of research^42^. In the recovery period, vasodilation^43^ and an alteration of the arterial baroreflex lead to a reduction of HR and sympathetic outflow compared to the exercise period^44–46^. These processes may have contributed to the fall of arterial pressure that we observed at the beginning of recovery. In concert with the HDT posture that facilitated venous return, this initial reduction of arterial pressure may have led to the observed transient increase of pulse BP. These cardiovascular responses, however, only lasted for less than 5 minutes: i.e. the values of sBP, dBP and MAP at the end of the recovery period (R_*late*_) were not different from baseline. This result suggests that the initial after-effects on the cardiovascular signals may have been preferentially caused by immediate postexercise hyperaemia^47^ and not by sustained vasodilation^48^.

Leg muscle loading influenced post-exercise cardiovascular responses at the beginning of the recovery phase. When subjects had to counteract the force applied by the leg-press device (HDT-ML), we observed slightly lower peak values of pulse BP and HR than in the sessions without muscle loading (HDT-noML) during early recovery R_*early*_ (Fig. 5). The reduction of pulse BP peak is at the limit of statistical significance, and therefore we cannot derive strong conclusions. However, this effect may be due to increased cardiac afterload, which is consistent both with the overall higher values of MAP and with the lower dBP undershoot observed when muscle loading was applied (Figs 3, 4 and 6). The reduction of the maximum value of HR may be due to a milder exercise-induced alteration of the carotid-cardiac baroreflex^40^ to keep blood pressure within safe ranges.

Our results have implications to the cardiovascular deconditioning that occurs during space missions. Similarly to HDT, microgravity causes a headward shift of body fluid that in the short term elicits a reduction of blood pressure and heart rate, and in the long term leads to a reduction of baroreflex sensitivity and ultimately to orthostatic intolerance^20–23^. In this study, we showed that loading leg muscles during HDT at rest causes an increase of BP that can establish similar values to those associated to orthostatic condition (i.e. earth-like). Similarly, dynamic exercises during HDT can temporarily increase HR and leg blood volume^49^. These findings motivate the development of lower limb wearable devices that can provide astronauts with leg muscle loading for extended periods of time, potentially limiting the reduction of blood pressure they experience in the microgravity environment. Long term bed-rest studies and experiments during space missions will be necessary to test whether such a sustained muscle loading, along with the intense sessions of dynamic exercises that astronauts perform regularly^27,28^, can ameliorate long-term effects of microgravity on the cardiovascular system.

Additional experiments will also be necessary to better understand the physiological bases of the findings reported here. A direct estimation of the baroreflex curves would allow us to quantify the influence of leg muscle loading on baroreflex characteristics during HDT. Introducing female subjects could elucidate possible gender-driven modulation of these mechanisms. Furthermore, applying different loading forces could reveal potential intensity-driven modulations. While previous experiments have already considered some of these aspects in orthostatic conditions^50–54^, the results may not directly translate to HDT as this condition is characterized by increased central blood volume and reduced venous pooling when compared to upright posture.

## Methods

### Participants

Seventeen male subjects (age: 29.7 ± 3.9 years, weight: 79.2 ± 7.7 kg, height: 1.79 ± 0.06 m, mean ± s.d.) volunteered to participate in this study, and signed an informed consent. Subjects had no history of cardiovascular nor musculoskeletal diseases, and were instructed to avoid caffeine and food before the experiment. All procedures were conducted in conformance with the Declaration of Helsinki and were approved by the Ethical Review Committee of Canton Zurich.

### Study protocol

The analyses reported in this paper are part of a larger study, which we describe here for completeness and reproducibility. After sensor placement and calibration, we recorded cardiovascular signals during orthostatic stress for 3 minutes (i.e. upright standing posture without movements; ORT condition). Then, we let the subjects lie on the tilted platform (HDT condition) and we secured them to the robotic leg-press device MARCOS. In this posture, they performed nine consecutive experimental sessions, each consisting of three phases: baseline, exercise, and recovery. During baseline, subjects kept their legs extended for 3 minutes. In preparation for the exercise phase, they flexed their legs until reaching the mechanical stop of MARCOS (20 seconds). During the exercise phase, they performed leg-press exercises against the resistance of the device. Finally during recovery, subjects kept their legs extended for 5 minutes.

The intensity of the exercises was defined as a combination of: (1) resistive force of the leg-press, F; (2) frequency of the leg-press movements, f; (3) duration of the exercise phase, T. We tested two values for each of these factors (see here^49^ for details), for a total of eight intensities and corresponding experimental sessions. In these sessions, MARCOS did not apply any force during baseline and recovery, so that no muscle contraction was needed to maintain leg extension (i.e. head-down tilt without leg muscle loading, HDT-noML). In an additional session, the device applied a force of ${\bar{F}}_{i}=g\times {w}_{i}/3$ N (where *w*_*i*_ is the weight of the *i*-th subject, and *g* = 9.6 *m*/*s*^2^ is the gravitational acceleration) during all the phases (i.e. head-down tilt with leg muscle loading, HDT-ML), and the leg-press exercises were performed at frequency f = 0.5 Hz and duration T = 30 s. The order of these nine sessions was randomized across subjects to avoid biases in the results.

For the purpose of this study, we considered the ORT condition, and only two of the HDT experimental sessions described above: HDT-ML, and HDT-noML with matching exercise intensity, i.e. F = ${\overline{F}}_{i}$, f = 0.5 Hz, T = 30 s. This allowed us to investigate the effects of leg muscle loading on the cardiovascular signals during HDT, both at rest (comparing the baseline phases of HDT-ML and HDT-noML, and using ORT as a reference) and during recovery from physical exercise (comparing the relative changes from baseline to recovery observed in HDT-ML and HDT-noML).

### MARCOS

MARCOS is a robotic device originally developed to execute leg movements inside a MRI scanner^29^. In this study, we adapted this device to perform subject-driven leg-press exercises on a 6° head-down tilted platform (Fig. 1c). Foot loading is realized by means of pneumatic actuators that can provide a simulated ground reaction force (i.e. opposing resistance to leg extension) of up to 400 N.

The user is secured to the platforms by means of: (1) shoes that are firmly attached to the foot pedals, (2) a brace that prevents mediolateral movements of the hip, and (3) shoulder straps that prevent sliding in the direction of force. The position of the brace as well as the length of the shoulder straps can be adjusted to the size of the user, allowing us to obtain very similar ranges of motion across subjects (hip: 0° to 40°; knee: 0° to 70°).

### Data acquisition and processing

Continuous blood pressure (BP) was measured by means of a CNAP® Monitor 500 (CNSystem Medizintechnik AG, Austria). This system uses the vascular unloading technique to estimate BP information from plethysmographic signals by means of two finger cuffs with integrated infrared light sensors^55^. In order to reduce motion artifacts, we used an adjustable strap to support the arm of the subject in a standardized position, allowing the hand to rest at the level of the heart.

The raw BP signal was low-pass filtered (10 Hz, 3rd order Butterworth). Systolic (sBP) and diastolic (dBP) traces were obtained by linear interpolation of the maximum and minimum peaks in the filtered BP wave respectively. Heart rate (HR) was computed as the ratio between 60 s and the time difference between adjacent systolic peaks. The obtained sBP, dBP and HR were further low pass filtered to evaluate their average trends along the experimental sessions (0.05 Hz, 3rd order Butterworth). Pulse blood pressure (pBP) was computed as pBP = sBP-dBP. Mean arterial pressure (MAP) was estimated as MAP = 1/3 sBP + 2/3 dBP.

From these continuous signals, we computed several features to characterize each phase of the experiment. During the orthostatic condition and the baseline phase of the HDT sessions, the signals were stable. Hence, we averaged each continuous trace over the last 90 s of recording. On the other hand, during the recovery phase, the signals exhibited an initial transient dynamics and then they stabilized. Therefore, we computed signal-specific features in the first 90 s of recovery (R_*early*_; see Sec. Results), and we averaged each signal over the last 90 s of recovery (R_*late*_).

Due to unexpected failures of the acquisition system during the recordings, we had to exclude one subject from the ORT condition and one subject from the HDT-ML condition, resulting in N = 16, 17, 16 subjects for ORT, HDT-noML, and HDT-ML respectively.

### Statistical analysis

We employed Linear Mixed Effect Models (LMEM) to analyze the cardiovascular signals both at baseline and during the recovery phase, using the nlme package^56^ in the R environment. LMEMs allowed us to consider variability at different levels of the dataset (e.g. across conditions and across subjects), and to cope with missing data, thus obtaining maximal statistical power from our dataset. To confirm that our dataset met the assumptions of Gaussian distribution and independence of residuals and random effects^57^, we visually inspected the distributions using qq-plots and histograms, and we employed Shapiro-Wilk Normality tests. After fitting the LMEMs, we tested our specific hypotheses of interest by performing post-hoc tests and using Bonferroni corrections to adjust the obtained p-values. Note that because of this correction, the adjusted p-values can be equal to 1. We considered tests to be statistically significant if their p-values were lower than the 0.05 significance level.

To assess the effect of leg muscle loading at rest and at the end of the recovery phase (R_*late*_), we fit a LMEM for each signal, using the average value of the signal across the 90 s window of interest as the dependent variable, and the following independent variables: subject weight (continuous variable), the phase of the experimental session (a factor with levels *baseline* and *recovery*), and the muscle loading condition (a factor with levels *ML* and *noML*). Finally, we considered the interaction term between phase and loading to analyze the potentially different effects of muscle loading at baseline (rest condition) and during R_*late*_. In order to take inter-subject variability into account, we considered subject identity as a random effect on the model intercept, obtaining a more powerful version of a repeated measure ANOVA. We then performed post-hoc tests to evaluate whether: (1) the baseline values of the signals with muscle loading were different from those without muscle loading; (2) the R_*late*_ values of the signals were different from baseline, both with and without muscle loading.

To evaluate the differences between the signals observed during orthostatic stress (ORT) and those recorded during head-down tilt at rest with and without muscle loading (HDT-ML and HDT-noML respectively), we fit a LMEM for each signal using the average value of the signal as the dependent variable, and the experimental condition as the independent variable. We also considered subject identity as a random effect on the model intercept. We then performed post-hoc tests to assess if the values of the signals obtained during each HDT condition were different from those obtained during ORT.

Finally, to evaluate the effect of muscle loading on the transient dynamics of the signals during early recovery (R_*early*_), we fit a LMEM for each signal using the signal-specific feature (see Sec. Results) as the dependent variable, and subject weight and muscle loading condition as the independent variables. We also considered subject identity as a random effect on the model intercept. We performed post-hoc tests to evaluate if the values of the features obtained with muscle loading were different from those obtained without muscle loading.

## Data Availability

The dataset and the code used for the current study are available in the OSF repository, https://osf.io/fgvh3/.

## References

[1] Sarabadani Tafreshi A, Riener R, Klamroth-Marganska V (2016). Distinctive steady-state heart rate and blood pressure responses to passive robotic leg exercise and functional electrical stimulation during head-up tilt. Front. Physiol..

[2] Sarabadani Tafreshi A, Riener R, Klamroth-Marganska V (2017). Distinctive steady-state heart rate and blood pressure responses to passive robotic leg exercise during head-up tilt: A pilot study in neurological patients. Front. Physiol..

[3] Shamsuzzaman AS, Sugiyama Y, Kamiya A, Fu Q, Mano T (1998). Head-up suspension in humans: effects on sympathetic vasomotor activity and cardiovascular responses. J. Appl. Physiol..

[4] Murphy MN, Mizuno M, Mitchell JH, Smith SA (2011). Cardiovascular regulation by skeletal muscle reflexes in health and disease. Am. journal physiology. Hear. circulatory physiology.

[5] Gladwell VF, Coote JH (2002). Heart rate at the onset of muscle contraction and during passive muscle stretch in humans: A role for mechanoreceptors. J. Physiol..

[6] Hartwich D, Dear WE, Waterfall JL, Fisher JP (2011). Effect of muscle metaboreflex activation on spontaneous cardiac baroreflex sensitivity during exercise in humans. J. Physiol..

[7] Verma, A. K. *et al*. Skeletal muscle pump drives control of cardiovascular and postural systems. *Sci. Reports* **7**, 10.1038/srep45301 (2017). 10.1038/srep45301 PMC5366896 28345674

[8] Krediet CT, Go-Schön IK, Van Lieshout JJ, Wieling W (2008). Optimizing squatting as a physical maneuver to prevent vasovagal syncope. Clin. Auton. Res..

[9] Krediet CT, Wilde AA, Halliwill JR, Wieling W (2005). Syncope during exercise, documented with continuous blood pressure monitoring during ergometer testing. Clin. Auton. Res..

[10] Watanabe N, Reece J, Polus BI (2007). Effects of body position on autonomic regulation of cardiovascular function in young, healthy adults. Chiropr. & Osteopathy.

[11] Kubota S, Endo Y, Kubota M, Shigemasa T (2017). Assessment of effects of differences in trunk posture during Fowler’s position on hemodynamics and cardiovascular regulation in older and younger subjects. Clin. Interv. Aging.

[12] Ogoh S (2006). Effects of changes in central blood volume on carotid-vasomotor baroreflex sensitivity at rest and during exercise. J. Appl. Physiol..

[13] Ray CA, Rea RF, Clary MP, Mark AL (1993). Muscle sympathetic nerve responses to dynamic one-legged exercise: Effect of body posture. Am. J. Physiol. - Hear. Circ. Physiol..

[14] Linnarsson D (1996). Blood pressure and heart rate responses to sudden changes of gravity during exercise. The Am. journal physiology.

[15] Prisk GK, Fine JM, Elliott AR, West JB (2002). Effect of 6 degrees head-down tilt on cardiopulmonary function: comparison with microgravity. Aviat. space, environmental medicine.

[16] Pavy-Le Traon, A., Heer, M., Narici, M. V., Rittweger, J. & Vernikos, J. *From space to Earth: advances in human physiology from 20 years of bed rest studies (1986–2006)*., vol. 101 (2007). 10.1007/s00421-007-0474-z 17661073

[17] Hargens AR, Vico L (2016). Long-duration bed rest as an analog to microgravity. J. Appl. Physiol..

[18] Shiraishi M, Schou M, Gybel M, Christensen NJ, Norsk P (2002). Comparison of acute cardiovascular responses to water immersion and head-down tilt in humans. J Appl Physiol (1985).

[19] Norsk P (1993). Volume-homeostatic mechanisms a 12-h posture change. J. applied physiology.

[20] Norsk P (2014). Blood pressure regulation IV: Adaptive responses to weightlessness. Eur. J. Appl. Physiol..

[21] Hughson, R. L., Helm, A. & Durante, M. Heart in space: effect of the extraterrestrial environment on the cardiovascular system. *Nat. Rev. Cardiol*., 10.1038/nrcardio.2017.157 (2017). 10.1038/nrcardio.2017.157 29053152

[22] Hargens AR, Richardson S (2009). Cardiovascular adaptations, fluid shifts, and countermeasures related to space flight. Respir. physiology & neurobiology.

[23] Tanaka K, Nishimura N, Kawai Y (2017). Adaptation to microgravity, deconditioning, and countermeasures. J. Physiol. Sci..

[24] Clement, G. *Fundamentals of Space Medicine*, vol. 17 arXiv:1011.1669v3 (2003).

[25] White RJ, Averner M (2001). Humans in space. Nat..

[26] Demontis GC (2017). Human pathophysiological adaptations to the space environment. Front. Physiol..

[27] Hargens AR, Bhattacharya R, Schneider SM (2013). Space physiology VI: Exercise, artificial gravity, and countermeasure development for prolonged space flight. Eur. J. Appl. Physiol..

[28] Trappe S (2009). Exercise in space: human skeletal muscle after 6 months aboard the International Space Station. J. applied physiology (Bethesda, Md.: 1985).

[29] Hollnagel C (2011). Brain activity during stepping: A novel MRI-compatible device. J. Neurosci. Methods.

[30] Marchal-Crespo L, Schneider J, Jaeger L, Riener R (2014). Learning a locomotor task: with or without errors? *J*. NeuroEngineering Rehabil..

[31] Pump B (2001). Central volume expansion is pivotal for sustained decrease in heart rate during seated to supine posture change. Am. journal physiology. Hear. circulatory physiology.

[32] Pump B, Kamo T, Gabrielsen A, Norsk P (2001). Mechanisms of hypotensive effects of a posture change from seated to supine in humans. Acta Physiol. Scand..

[33] Charkoudian N (2004). Influence of increased central venous pressure on baroreflex control of sympathetic activity in humans. Am. journal physiology. Hear. circulatory physiology.

[34] Shi X, Foresman BH, Raven PB (1997). Interaction of central venous pressure, intramuscular pressure, and carotid baroreflex function. The Am. journal physiology.

[35] Schwartz, C. E. & Stewart, J. M. The arterial baroreflex resets with orthostasis. *Front. Physiol*. **3**, 10.3389/fphys.2012.00461 (2012). 10.3389/fphys.2012.00461 PMC3516802 23233840

[36] Williamson JW, Fadel PJ, Mitchell JH (2006). New insights into central cardiovascular control during exercise in humans: A central command update. Exp. Physiol..

[37] Matsukawa K (2012). Central command: Control of cardiac sympathetic and vagal efferent nerve activity and the arterial baroreflex during spontaneous motor behaviour in animals. Exp. Physiol..

[38] Kaufman MP (2012). The exercise pressor reflex in animals. Exp. Physiol..

[39] Secher NH, Amann M (2012). Human investigations into the exercise pressor reflex. Exp. Physiol..

[40] Michelini LC, O’Leary DS, Raven PB, Nóbrega ACL (2015). Neural control of circulation and exercise: a translational approach disclosing interactions between central command, arterial baroreflex, and muscle metaboreflex. Am. J. Physiol. - Hear. Circ. Physiol..

[41] Raven PB, Fadel PJ, Ogoh S (2006). Arterial baroreflex resetting during exercise: A current perspective. Exp. Physiol..

[42] Romero SA, Minson CT, Halliwill JR (2017). The cardiovascular system after exercise. J. Appl. Physiol..

[43] Halliwill JR, Buck TM, Lacewell AN, Romero SA (2013). Postexercise hypotension and sustained postexercise vasodilatation: What happens after we exercise?. Exp. Physiol..

[44] Halliwill JR, Taylor JA, Eckberg DL (1996). Impaired sympathetic vascular regulation in humans after acute dynamic exercise. The J. Physiol..

[45] Seiler, S., Haugen, O. & Kuffel, E. Autonomic recovery after exercise in trained athletes: Intensity and duration effects. *Medicine Sci. Sports Exerc*. **39**, 1366–1373, 10.1249/mss.0b013e318060f17d1111.6189v1 (2007). 10.1249/mss.0b013e318060f17d 17762370

[46] Chen C-Y, Bonham AC (2010). Postexercise hypotension: central mechanisms. Exerc. sport sciences reviews.

[47] Bangsbo J, Hellsten Y (1998). Muscle blood flow and oxygen uptake in recovery from exercise. Acta physiologica Scand..

[48] McCord JL, Halliwill JR (2006). H1 and H2 receptors mediate postexercise hyperemia in sedentary and endurance exercise-trained men and women. J. Appl. Physiol..

[49] Alessandro, C., Tafreshi, A. S. & Riener, R. Increasing leg blood volume during head-down tilt by performing physical exercises, a preliminary study. In *2016 6th IEEE International Conference on Biomedical Robotics and Biomechatronics (BioRob)*, 888–893, 10.1109/BIOROB.2016.7523740 (Singapore, 2016).

[50] Queiroz AC (2013). Gender influence on post-resistance exercise hypotension and hemodynamics. Int. J. Sports Medicine.

[51] Senitko AN, Charkoudian N, Halliwill JR (2002). Influence of endurance exercise training status and gender on postexercise hypotension. J. Appl. Physiol..

[52] Brito LC, Queiroz AC, Forjaz CL (2014). Influence of population and exercise protocol characteristics on hemodynamic determinants of post-aerobic exercise hypotension. Braz. J. Med. Biol. Res..

[53] Fadel PJ, Raven PB (2012). Human investigations into the arterial and cardiopulmonary baroreflexes during exercise. Exp. Physiol..

[54] Arzeno NM, Stenger MB, Lee SMC, Ploutz-Snyder R, Platts SH (2013). Sex differences in blood pressure control during 6 degrees head-down tilt bed rest. Am. J. Physiol. Hear. Circ. Physiol..

[55] Fortin J (2006). Continuous non-invasive blood pressure monitoring using concentrically interlocking control loops. Comput. Biol. Medicine.

[56] Pinheiro, J., Bates, D., DebRoy, S., Sarkar, D. & R Core Team. nlme: Linear and Nonlinear Mixed Effects Models (2017).

[57] Pinheiro, J. C. & Bates, D. M. *Mixed effects models in S and S-Plus* 9809069v1 (Springer, 2000).

